# Adaptor protein CrkII negatively regulates osteoblast differentiation and function through JNK phosphorylation

**DOI:** 10.1038/s12276-019-0314-3

**Published:** 2019-09-25

**Authors:** Jung Ha Kim, Kabsun Kim, Inyoung Kim, Semun Seong, Kwang-Il Nam, Kyung Keun Kim, Nacksung Kim

**Affiliations:** 1 Department of Pharmacology, Chonnam National University Medical School, Gwangju, 61469 Republic of Korea; 2 Department of Biomedical Sciences, Chonnam National University Medical School, Gwangju, 61469 Republic of Korea; 3 Department of Anatomy, Chonnam National University Medical School, Gwangju, 61469 Republic of Korea

**Keywords:** Cell signalling, Transcription

## Abstract

The adaptor protein CrkII is involved in several biological activities, including mitogenesis, phagocytosis, and cytoskeleton reorganization. Previously, we demonstrated that CrkII plays an important role in osteoclast differentiation and function through Rac1 activation both in vitro and in vivo. In this study, we investigated whether CrkII also regulates the differentiation and function of another type of bone cells, osteoblasts. Overexpression of CrkII in primary osteoblasts inhibited bone morphogenetic protein (BMP) 2-induced osteoblast differentiation and function, whereas knockdown of CrkII expression exerted the opposite effect. Importantly, CrkII strongly enhanced c-Jun-N-terminal kinase (JNK) phosphorylation, and the CrkII overexpression-mediated attenuation of osteoblast differentiation and function was recovered by JNK inhibitor treatment. Furthermore, transgenic mice overexpressing CrkII under control of the alpha-1 type I collagen promoter exhibited a reduced bone mass phenotype. Together, these results indicate that CrkII negatively regulates osteoblast differentiation and function through JNK phosphorylation. Given that CrkII acts as a negative and positive regulator of osteoblast and osteoclast differentiation, respectively, the regulation of CrkII expression in bone cells may help to develop new strategies to enhance bone formation and inhibit bone resorption.

## Bone metabolism: Adaptor protein prevents bone formation

A protein that facilitates interactions between proteins and regulates cell signalling pathways blocks the differentiation and function of bone-forming osteoblast cells. Nacksung Kim and colleagues at Chonnam National University Medical School in Gwangju, South Korea, previously showed that CrkII, a so-called adaptor protein that links protein-binding partners and enables the formation of larger signaling complexes, enhanced the differentiation of bone-resorbing osteoclasts and stimulated their resorptive function. Now, they demonstrate in cells and transgenic mice that CrkII also prevents the formation of osteoblasts from precursor cells and reduces osteoblast activity. This effect involved the activation of two specific signalling proteins and was reversed by their inhibitors. Therefore, CrkII could be a useful target for both stimulating bone formation and reducing resorption.

## Introduction

Osteoblasts are mononuclear cells that are responsible for bone formation. The differentiation of mesenchymal cells into osteoblasts is regulated by various anabolic factors, such as bone morphogenetic proteins (BMPs), insulin, and insulin-like growth factor-1^1–3^. Notably, BMPs, which belong to the transforming growth factor-β (TGF-β) superfamily of proteins, were initially discovered to induce bone formation. Binding of BMPs to their type I and II serine/threonine kinase receptors activates the Smad signaling pathway, inducing the expression of various target genes, such as runt-related transcription factor 2 (Runx2), alkaline phosphatase (ALP), and osteocalcin^4–6^.

Various transcription factors involved in osteoblast differentiation and function, such as the Runx2, osterix, and homeodomain-containing Msx proteins, have been well studied^1,7–10^. In particular, Runx2 is a master transcription factor that regulates the expression of many osteoblastic marker genes, including ALP, bone sialoprotein (BSP), and osteocalcin to induce osteoblast differentiation and bone formation^11,12^. Runx2, also known as Cbfa1, belongs to the runt homology domain protein family and contains a glutamine/alanine-rich domain at its N-terminal end (a runt domain) and a proline/serine/threonine-rich region at its C-terminus^11^. Runx2 has been shown to be an essential transcription factor for osteoblast differentiation and mineralization in multiple in vitro and in vivo studies. Mice with a homozygous mutation in Runx2 exhibit a complete lack of bone formation due to ossification failure^13,14^. Furthermore, mice with a heterozygous mutation in Runx2 exhibit cleidocranial dysplasia disorders associated with defective endochondral and intramembranous bone formation^13,15^. In contrast, transgenic mice overexpressing Runx2 show an increase in mineralizing bone surface, mineral apposition rate, and bone formation. Furthermore, the ectopic expression of Runx2 in even nonosteoblastic cells induces the expression of major osteoblastic marker genes^16^.

Recently, mitogen-activated protein kinase (MAPK) cascades, including the c-Jun-N-terminal kinase (JNK), extracellular signal-regulated kinase (Erk), and p38 cascades, have been revealed as noncanonical pathways for BMP2 signal transduction^17–20^. MAPKs can control osteoblast differentiation through the regulation of Runx2 phosphorylation and transcriptional activity. Runx2 phosphorylation by both Erk and p38 promotes BMP2-induced osteoblast differentiation^21,22^. In contrast, Runx2 phosphorylation by JNK inhibits osteoblast differentiation via the negative regulation of Runx2 function^6^.

CrkII belongs to a family of adaptor proteins that includes CrkI, CrkII, CrkIII, and CrkL^23^. The Crk family adaptor proteins predominantly contain Src homology 2 (SH2) and SH3 protein-binding domains^24,25^. Notably, CrkII contains one N-terminal SH2 domain and two SH3 domains (nSH3 and cSH3). Although it lacks an enzymatic kinase domain, CrkII plays a central role in several biological activities, such as mitogenesis, phagocytosis, and cytoskeleton reorganization, through constructing distinct combinations of protein interactions^26–29^. In our previous study, we demonstrated that CrkII plays critical roles in osteoclast differentiation and function through the regulation of Rac1 activity^23^. Since CrkII is ubiquitously expressed in most tissues, in the present study, we investigated the potential role of CrkII in osteoblast differentiation and function to clarify its role in bone homeostasis.

## Materials and methods

### Osteoblast differentiation

The Committee of Ethics in Animal Research approved all animal procedures performed in this study (approval number: CNU IACUC-H-2017-27). Primary osteoblast precursor cells were isolated from neonatal ICR mouse (Dae Han BioLink Co., Korea) skulls by enzymatic digestion using 0.1% collagenase (Life Technologies, Carlsbad, CA) and 0.2% dispase II (Roche Diagnostics GmbH, Mannheim, Germany). The cells were cultured in osteogenic medium (OGM) containing BMP2 (100 ng/mL), ascorbic acid (50 μg/mL), and β-glycerophosphate (100 mM) for osteoblast differentiation. Osteoblast precursor cells cultured for 3 days were used for ALP activity analysis to evaluate osteoblast differentiation. Cells were lysed using an osteoblast lysis buffer [50 mM Tris-HCl (pH 7.4), 1% Triton X-100, 150 mM NaCl, and 1 mM EDTA]. Cell lysates were incubated with *p*-nitrophenyl phosphate substrate (Sigma-Aldrich, St Louis, MO), and ALP activity was measured by measuring the absorbance at 405 nm using a spectrophotometer. To evaluate mineralization, cells were cultured for 9–12 days, fixed with 70% ethanol, and stained with 40 mM alizarin red (pH 4.2). After nonspecific staining was removed with a phosphate-buffered saline wash, alizarin red staining was visualized with a CanoScan 4400F scanner (Canon Inc., Japan). To quantify substrate calcification, alizarin red-stained cells were incubated with a 10% cetylpyridinium chloride solution for 30 min at room temperature, and the absorbance at 562 nm was measured.

### Retroviral gene transduction

Retroviral vectors were transfected into the Plat-E packaging cell line using FuGENE 6 (Promega, Madison, WI) according to the manufacturer’s protocol. Viral supernatants were recovered from the culture medium at 48 h after transfection. Osteoblasts were incubated with viral supernatants for 6 h in the presence of 10 μg/mL polybrene (Sigma-Aldrich).

### Reverse transcription-polymerase chain reaction

Total RNA was extracted from cultured cells using Qiazol lysis reagent (Qiagen, Hilden, Germany) according to the manufacturer’s instructions, and 2 μg of RNA was reverse transcribed into cDNA using SuperScript II Reverse Transcriptase (Life Technologies). To assess mRNA expression levels, the cDNA was amplified by polymerase chain reaction (PCR) using specific primers. The primer sequences were as follows: glyceraldehyde-3-phosphate dehydrogenase (GAPDH), 5ʹ-TGA CCA CAG TCC ATG CCA TCA CTG-3ʹ and 5ʹ-CAG GAG ACA ACC TGG TCC TCA GTG-3ʹ; Crk, 5ʹ-AGG CAG GGT AGT GGA GTG ATT CTC A-3ʹ and 5ʹ-CAT CTG TCA GCA AAC TGT CGA GCT AT-3ʹ; CrkIII, 5ʹ-AGG CAG GGT AGT GGA GTG ATT CTC A-3ʹ and 5ʹ-CGT TTC GCT GTA TCA GCA TTC CTA C-3ʹ; and CrkL, 5ʹ-GTG TCT CGC ACT ACA TCA TCA A-3ʹ and 5ʹ-GCT GAG ACA GAA CCC ACT GG-3ʹ.

### Real-time PCR

Quantitative real-time PCR analyses were performed in triplicate on a Rotor-Gene Q (Qiagen) using SYBR Green PCR Master Mix (Qiagen). Target gene expression was normalized to the level of an endogenous control, GAPDH. The relative quantitative value of each target gene compared with the calibrator for that target was expressed as 2^–(Ct–Cc)^, where Ct and Cc represent the mean threshold cycle differences after normalization to GAPDH. The relative expression levels of the samples are presented in a semilog plot. The primer sequences were as follows: GAPDH, 5ʹ-TGA CCA CAG TCC ATG CCA TCA CTG-3ʹ and 5ʹ-CAG GAG ACA ACC TGG TCC TCA GTG-3ʹ; Crk, 5ʹ-AGG CAG GGT AGT GGA GTG ATT CTC A-3ʹ and 5ʹ-CAT CTG TCA GCA AAC TGT CGA GCT AT-3ʹ; Runx2, 5ʹ-CCC AGC CAC CTT TAC CTA CA-3ʹ and 5ʹ-CAG CGT CAA CAC CAT CAT TC-3ʹ; ALP, 5ʹ-CAA GGA TAT CGA CGT GAT CAT G-3ʹ and 5ʹ-GTC AGT CAG GTT GTT CCG ATT C-3ʹ; BSP, 5ʹ-GGA AGA GGA GAC TTC AAA CGA AG-3ʹ and 5ʹ-CAT CCA CTT CTG CTT CTT CGT TC-3ʹ; and Osteocalcin, 5ʹ-ATG AGG ACC CTC TCT CTG CTG CTC AC-3ʹ and 5ʹ-AGA GCA AAC TGC AGA AGC TGA GAG-3ʹ.

### Short interfering RNA (siRNA) transfection

Control, Crk, and CrkL siRNAs were purchased from Dharmacon (Lafayette, CO) and transfected into osteoblasts using Lipofectamine 2000 (Life Technologies) according to the manufacturer’s protocol.

### Western blotting

Cultured cells were lysed in extraction buffer [50 mM Tris-HCl (pH 8.0), 150 mM NaCl, 1 mM EDTA, 0.5% Nonidet P-40, 1 mM PMSF, and protease inhibitor cocktail] and quantified using a BCA Protein Assay kit (Pierce, Rockford). Equal amounts of protein were subjected to sodium dodecyl sulfate-polyacrylamide gel electrophoresis and transferred to a polyvinylidene fluoride membrane (Millipore, Billerica, MA). Membranes were incubated with appropriate primary antibodies against Flag, actin (Sigma-Aldrich), Runx2 (Santa Cruz Biotechnology, Santa Cruz, CA), phospho-Smad, Smad, phospho-p38, p38, phospho-JNK, JNK, phospho-Erk, Erk (Cell Signaling Technology, Beverly, MA), and lamin B1 (Abchem, Cambridge, UK). Following washing and incubation with appropriate horseradish peroxidase-linked secondary antibodies, signals were detected with an LAS3000 luminescent image analyzer (GE Healthcare, Piscataway, NJ).

### Generation of CrkII transgenic mice

Wild-type CrkII cDNA was fused to the mouse Col1α1 promoter. Transgenic mice were generated by standard pronuclear injection into C57BL/6 mice. Genomic DNA was isolated from the tail, and mice were genotyped using PCR. The following primer sequences were used: 5ʹ-GAG TTT TCG GAG ACG CTA AGC ACA TAG-3ʹ and 5ʹ-GAG TTT TCG GAG ACG CTA AGC ACA TAG-3ʹ.

### Microcomputed tomography analysis

Distal femurs were isolated from 8-week-old wild type or CrkII transgenic mice, dissected, and fixed overnight in 75% ethanol. For microcomputed tomography (μCT) analyses, the femurs were scanned using a high-resolution SkyScan 1172 system (SkyScan, Kontich, Belgium) at 50 kV and 201 μA with a 0.5 mm aluminum filter at a resolution of 11 μm pixel^−1^. Images were captured every 0.7° over an angular range of 180°. Raw images were reconstructed into serial cross-sections, and femoral morphometric parameters were analyzed using image reconstruction software (NRecon 1.4; SkyScan), data analysis software (CTAn; SkyScan), and three-dimensional model visualization software (Ant 2.4; SkyScan).

### Bone histomorphometric analysis

Tibiae were collected from 8-week-old wild-type or CrkII transgenic mice, fixed overnight in 4% paraformaldehyde, and decalcified in 5.5% EDTA buffer for 2 weeks at 4 °C. The decalcified tibiae were processed for paraffin embedding, and 4-μm-thick longitudinal sections were prepared. Bone sections were deparaffinized, hydrated, and stained with hematoxylin and eosin (H&E) or tartrate-resistant acid phosphatase.

### Statistical analysis

All values are expressed as the mean ± SD. Statistically significant differences were determined using a two-tailed Student’s *t-*test for two independent samples. Differences with *p* values less than 0.05 were considered statistically significant. Comparisons of three or more samples were analyzed by one-way analysis of variance.

## Results

### CrkII negatively regulates osteoblast differentiation and function

**Fig. 1:**
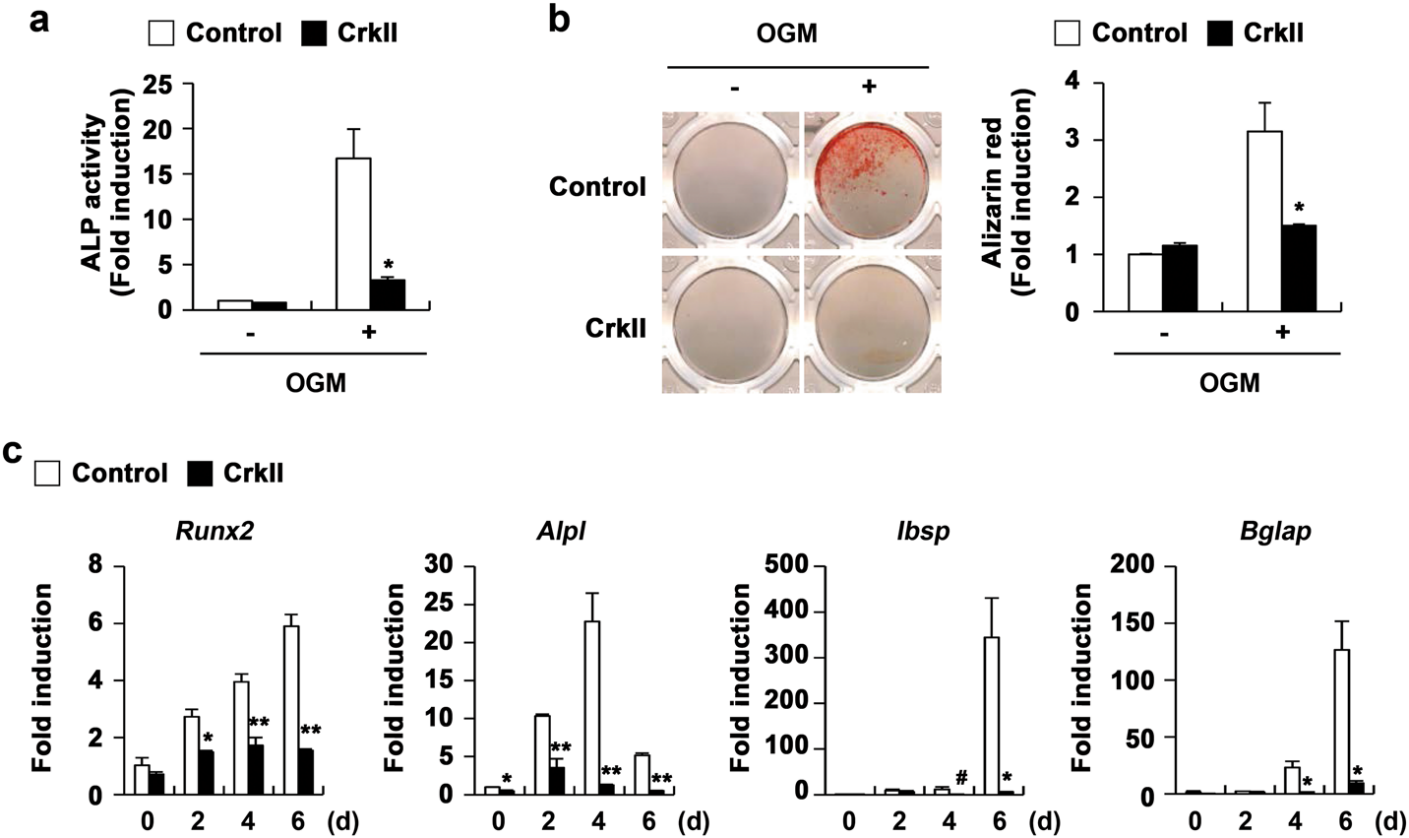
CrkII overexpression inhibits osteoblast differentiation and function. **a–c** Primary osteoblast precursor cells were transduced with pMX-IRES-EGFP (control) or CrkII retrovirus and cultured in OGM containing BMP2, ascorbic acid, and β-glycerophosphate. **a** Cells were cultured for 3 days and subjected to an ALP activity assay. **b** Cells were cultured for 9 days, fixed, and stained with alizarin red (left panel). Staining intensities were quantified at 562 nm via densitometry (right panel). **c** Total RNA was collected at each indicated time point, and real-time PCR was performed to evaluate the expression of the target genes. Data are expressed as the mean ± SD of triplicate samples. ^#^*p* < 0.05, *p < 0.01, **p *<* 0.001 versus control

When we examined the expression levels of Crk family members in osteoblasts, all Crk family members, including CrkI, CrkII, CrkIII, and CrkL, were expressed continuously during osteoblast differentiation (Fig. S1). To determine the role of CrkII in osteoblast differentiation and function, control- or CrkII-transduced osteoblasts were cultured in OGM, and the ALP activity and alizarin red staining intensities were then quantified. ALP activity was strongly induced by OGM stimulation, and this induction was greatly reduced in CrkII-overexpressing cells (Fig. 1a). Similarly, bone mineralization measured by quantifying the alizarin red staining intensity was also inhibited in CrkII-overexpressing cells compared with that in control cells (Fig. 1b). Consistent with changes in ALP activity and bone mineralization, the expression of the typical osteogenic marker genes *Runx2*, *Alpl*, *Ibsp*, and *Bglap* was significantly inhibited by CrkII overexpression (Fig. 1c).

**Fig. 2:**
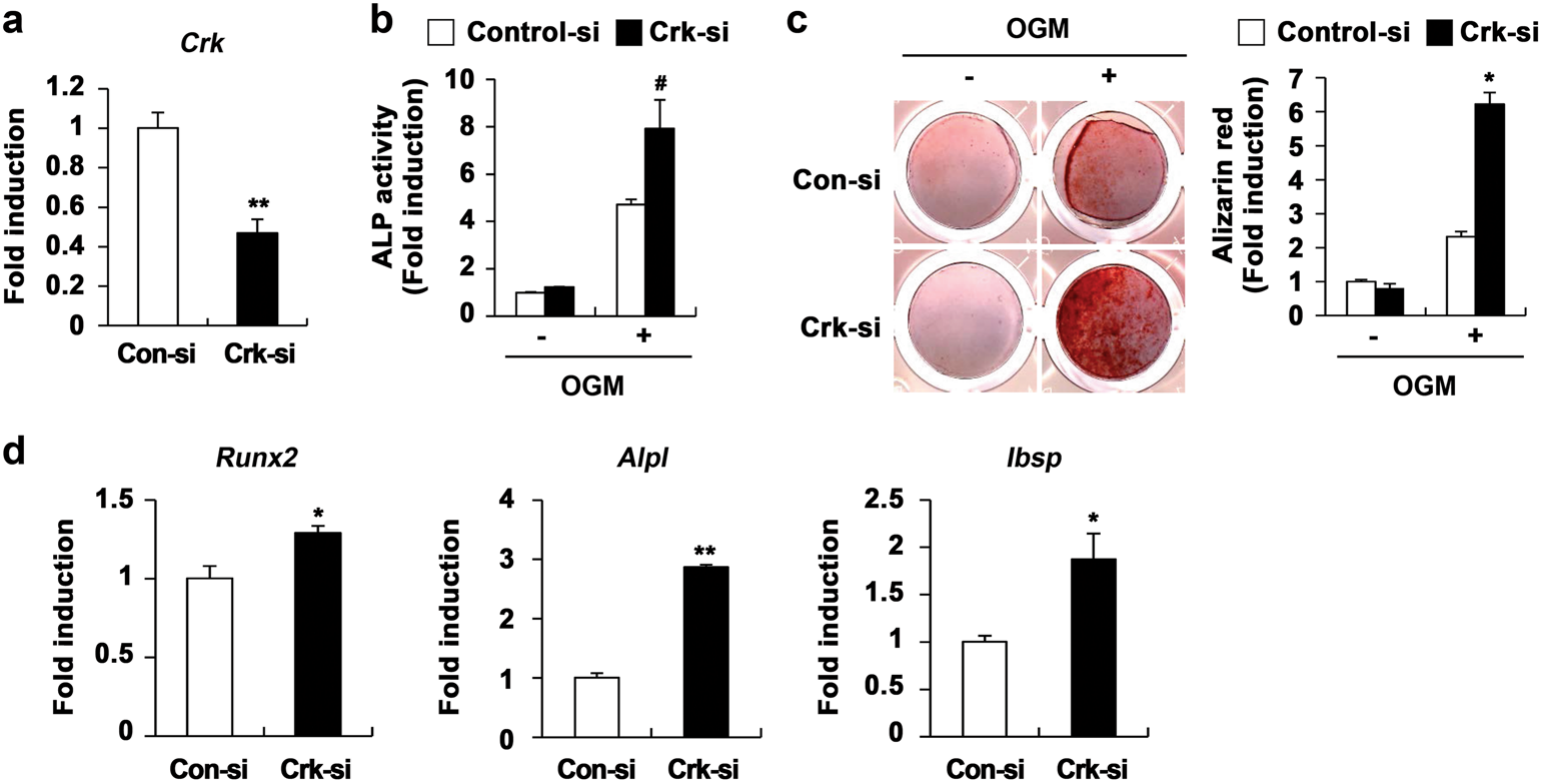
CrkII downregulation enhances osteoblast differentiation and function. **a** Control siRNA- or Crk siRNA-transfected osteoblasts were cultured for 2 days. Real-time PCR was performed to assess CrkII mRNA expression. **b–d** Osteoblasts were transfected with control siRNA or Crk siRNA and cultured in OGM. **b** Cells were cultured for 3 days and subjected to an ALP activity assay. **c** Cells were cultured for 9 days, fixed, and stained with alizarin red (left panel). Staining intensities were quantified at 562 nm via densitometry (right panel). **d** Total RNA was collected at each indicated time point, and real-time PCR was performed to evaluate the expression of the target genes. Data are expressed as the mean ± SD of triplicate samples. ^#^*p* < 0.05, *p < 0.01, **p *<* 0.001 versus control

To confirm the role of CrkII in osteoblast differentiation and function, osteoblasts were transfected with siRNAs targeting CrkII. Downregulation of endogenous CrkII expression (Fig. 2a) resulted in increased ALP activity, bone mineralization, and expression of typical osteogenic marker genes (Fig. 2b–d). Collectively, these data suggest that CrkII negatively regulates osteoblast differentiation and function.

### CrkII and CrkL have overlapping functions in osteoblasts

**Fig. 3:**
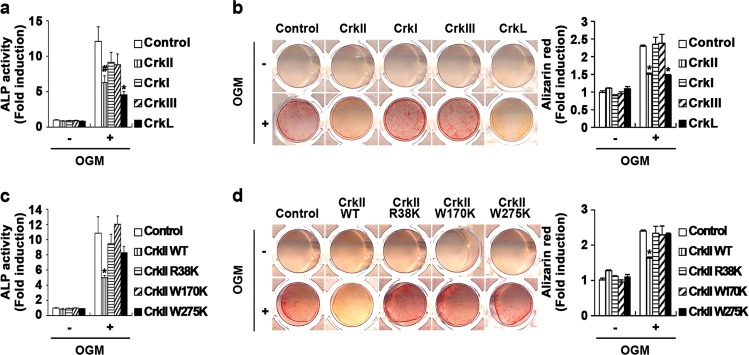
The roles of the three SH domains in CrkII in osteoblasts. **a–b** Osteoblasts were transduced with pMX-IRES-EGFP (control), CrkII, CrkI, CrkIII, or CrkL retrovirus. Transduced osteoblasts were cultured in OGM. **a** Cells were cultured for 3 days and subjected to an ALP activity assay. **b** Cells were cultured for 9 days, after which they were fixed and stained with alizarin red (left panel). Staining intensities were quantified at 562 nm via densitometry (right panel). **c–d** Osteoblasts were transduced with pMX-IRES-EGFP (control), CrkII-WT, CrkII-R38K, CrkII-W170K, or CrkII-W275K retrovirus. Transduced osteoblasts were cultured in OGM. **c** Cells were cultured for 3 days and subjected to an ALP activity assay. **d** Cells were cultured for 9 days, after which they were fixed and stained with alizarin red (left panel). Staining intensities were quantified at 562 nm via densitometry (right panel). Data are expressed as the mean ± SD of triplicate samples. ^#^*p* < 0.05, *p < 0.01 versus control

Since all Crk family adaptor proteins were expressed in osteoblasts (Fig. S1), we investigated the roles of other Crk family adaptors in osteoblasts. As shown in Fig. 3a, b, similar to the effects of CrkII overexpression, the overexpression of only CrkL significantly inhibited ALP activity and bone mineralization. CrkL overexpression also reduced the expression of typical osteogenic marker genes (Fig. S2). Furthermore, downregulation of both CrkII and CrkL synergistically increased bone mineralization (Fig. S3). These results indicated that among the Crk family adaptors, CrkII and CrkL, but not CrkI and CrkIII, inhibit osteoblast differentiation and function.

Although CrkL is the product of a distinct gene, it contains one SH2 domain and two SH3 domains like CrkII, whereas CrkI lacks the cSH3 domain and regulatory tyrosine phosphorylation site, and CrkIII contains a partially disrupted cSH3 domain^24,25,30–32^. Therefore, we tested whether three SH domains are required for the function of CrkII in osteoblasts. Overexpression of CrkII significantly inhibited ALP activity and bone mineralization, but overexpression of R38K CrkII (mutated in SH2), W170K CrkII (mutated in nSH3), and W275K CrkII (mutated in cSH3) did not inhibit ALP activity or bone mineralization (Fig. 3c, d). These data suggest that the three SH domains in CrkII are required for CrkII-regulated osteoblast differentiation and function.

### CrkII regulates osteoblast differentiation and function via JNK activation

**Fig. 4:**
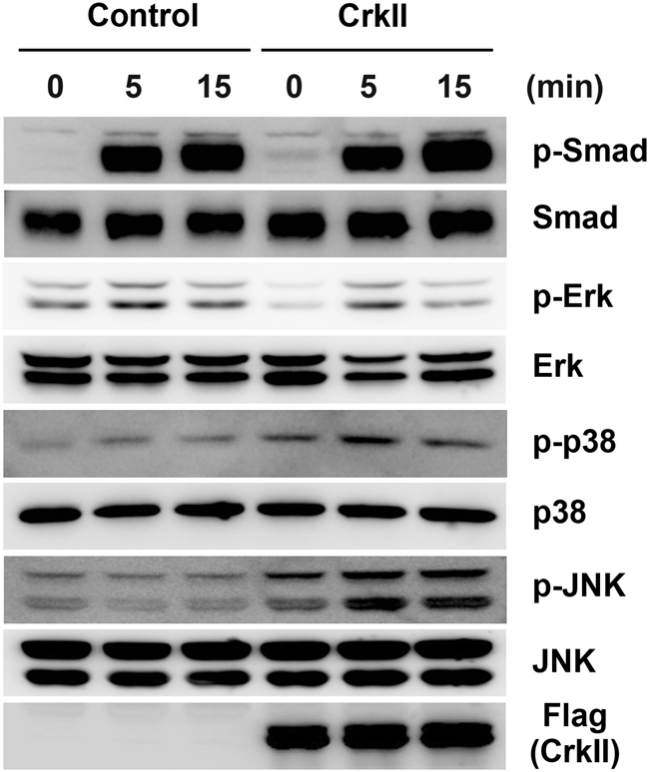
CrkII increases JNK phosphorylation. Osteoblasts were transduced with pMX-IRES-EGFP (control) or CrkII retrovirus and stimulated with BMP2 for the indicated times. Whole-cell lysates were analyzed by western blotting using specific antibodies, as indicated

Next, we determined which signaling pathway is most responsible for the CrkII-induced inhibition of osteoblast differentiation and function. As shown in Fig. 4, BMP2 stimulation successfully induced the phosphorylation of Smad1/5/8, Erk, and p38, which positively regulate osteoblast differentiation^33–37^. When CrkII was overexpressed in osteoblasts, the BMP2-induced phosphorylation of p38 was slightly increased, but that of Smad1/5/8 and Erk was not. Interestingly, in control cells, BMP2 did not induce the phosphorylation of JNK, which negatively regulates osteoblast differentiation^6^, whereas the phosphorylation of JNK was increased in CrkII-overexpressing cells and even more strongly increased by BMP2 stimulation (Fig. 4).

**Fig. 5:**
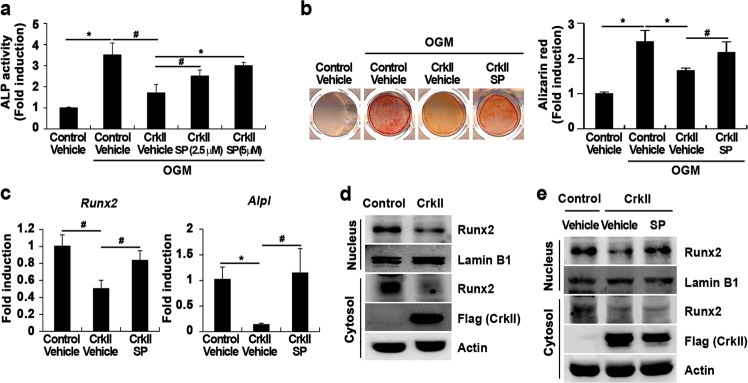
CrkII regulates osteoblast differentiation through enhancing JNK activation. **a–e** Osteoblasts were transduced with pMX-IRES-EGFP (control) or CrkII retrovirus. Transduced osteoblasts were treated with vehicle or SP600125 (2.5 or 5 µM) and cultured in OGM. **a** Cells were cultured for 3 days and subjected to an ALP activity assay. **b** Cells were cultured for 9 days, after which they were fixed and stained with alizarin red (left panel). Staining intensities were quantified at 562 nm via densitometry (right panel). **c** Total RNA was collected from cells cultured for 4 days, and real-time PCR was performed to evaluate expression of the target genes. **d**, **e** Cytoplasmic and nuclear fractions were harvested from cultured cells and subjected to western blot analysis using specific antibodies, as indicated. Data are expressed as the mean ± SD of triplicate samples. ^#^*p* < 0.05, *p < 0.01 versus control

To examine the correlation between reduced osteoblast differentiation and increased p38 phosphorylation by CrkII, we used a specific p38 MAPK inhibitor, SB203580. As shown in Fig. S4, treatment with SB203580 did not affect the CrkII-induced reduction in ALP activity, indicating that CrkII regulates osteoblast differentiation and function through signaling pathways other than the p38 MAPK pathway. Next, we tested whether CrkII inhibits osteoblast differentiation and function through activation of the JNK pathway. Treatment with a specific JNK inhibitor, SP600125, increased ALP activity, bone mineralization, and the expression of osteogenic marker genes reduced by CrkII overexpression (Fig. 5a–c). Therefore, these results indicated that CrkII regulates osteoblast differentiation and function through enhancing activation of the JNK pathway. To further examine the correlation between reduced osteoblast differentiation and increased JNK activation by CrkII, we investigated the effects of CrkII and SP600125 on Runx2 translocation. Importantly, CrkII inhibited the nuclear translocation of Runx2, and its effects were attenuated by the inhibition of JNK activity (Fig. 5d, e). Collectively, these results indicate that activation of the JNK pathway by CrkII reduces osteoblast differentiation and function by inhibiting the nuclear translocation of Runx2.

**Fig. 6:**
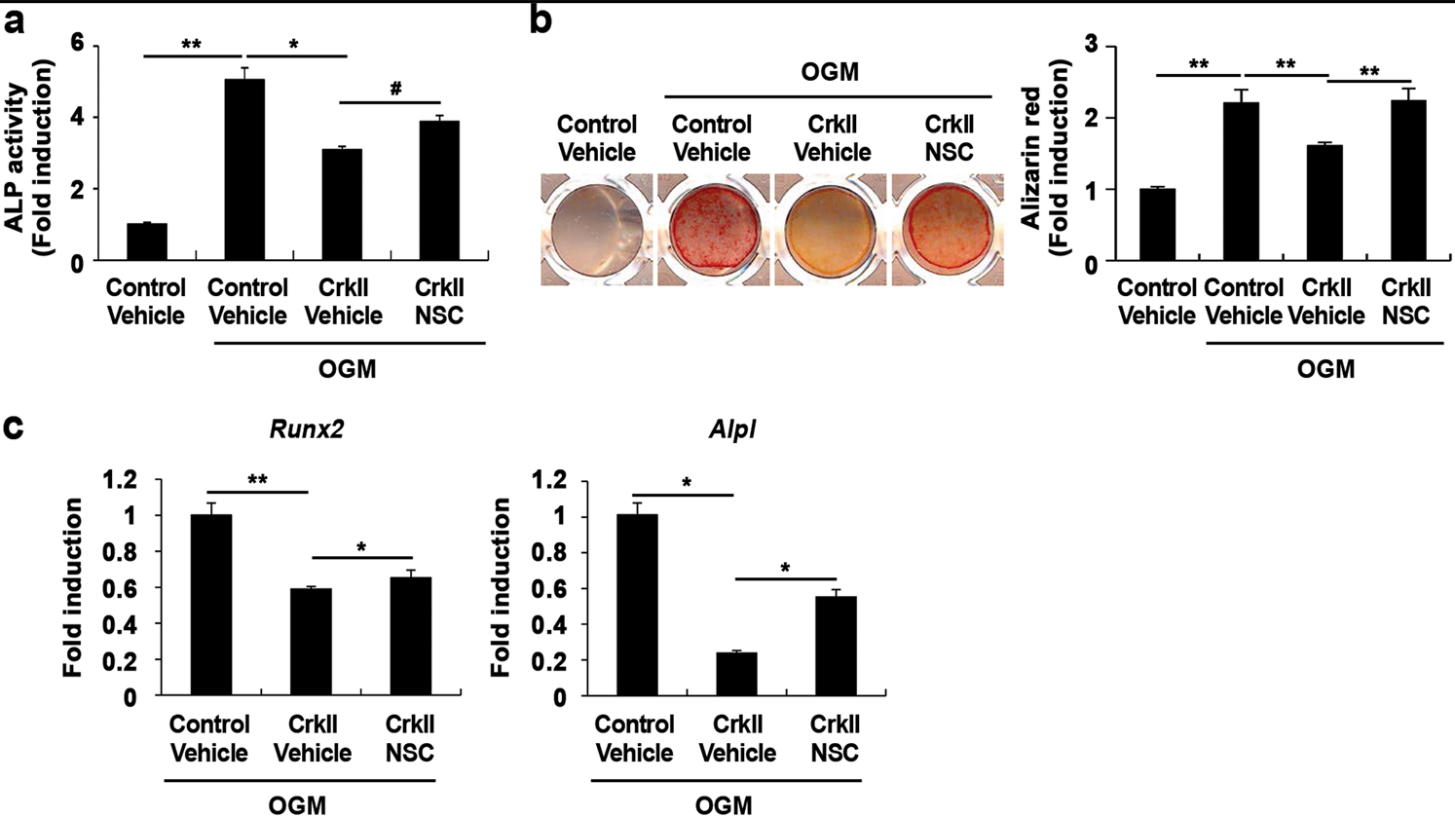
CrkII regulates osteoblast differentiation via Rac1 activation. **a–c** Osteoblasts were transduced with pMX-IRES-EGFP (control) or CrkII retrovirus. Transduced osteoblasts were treated with vehicle or NSC23766 (50 µM) and cultured in OGM. **a** Cells were cultured for 3 days and subjected to an ALP activity assay. **b** Cells were cultured for 9 days, after which they were fixed and stained with alizarin red (left panel). Staining intensities were quantified at 562 nm via densitometry (right panel). **c** Total RNA was collected from cells cultured for 4 days, and real-time PCR was performed to evaluate expression of the target genes. Data are expressed as the mean ± SD of triplicate samples. ^#^*p* < 0.05, *p < 0.01, **p *<* 0.001 versus control

We previously reported that the role of CrkII in osteoclast differentiation and function is linked to Rac1 activation^23^. Moreover, BMP2-induced Rac1 activation has been shown to inhibit osteoblast differentiation^38^. Therefore, we examined whether the negative role of CrkII in osteoblast differentiation is associated with Rac1 activation. Treatment with a specific Rac1 inhibitor, NSC23766, increased ALP activity, bone mineralization, and expression of osteogenic marker genes reduced by CrkII overexpression (Fig. 6a–c), indicating that the negative role of CrkII in osteoblast differentiation is linked to Rac1 activation.

### Transgenic mice overexpressing CrkII in osteoblasts exhibit low bone mass

**Fig. 7:**
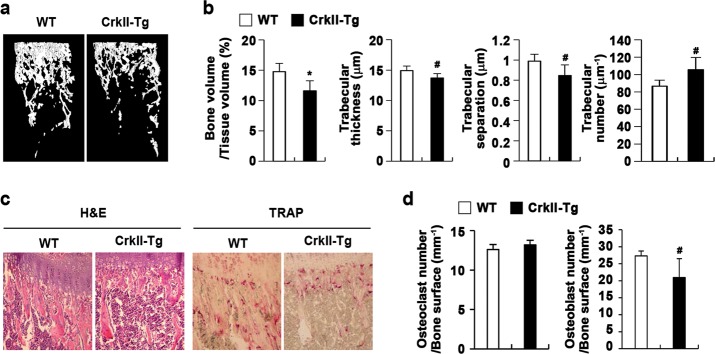
CrkII transgenic mice exhibit low bone mass. **a** Representative three-dimensional images of the femurs from CrkII transgenic mice and their wild-type littermates. **b** Bone volume to tissue volume and the trabecular bone thickness, separation, and number were assessed using μCT measurements (*n* = 7 or 5). **c** H&E and TRAP staining of histological sections of proximal tibiae. **d** The numbers of osteoblasts and osteoclasts per bone surface area were assessed (*n* = 5 or 4). ^#^*p* < 0.05, *p < 0.01 versus control

After the inhibitory roles of CrkII in osteoblast differentiation and function were confirmed, to further investigate the effects of CrkII on bone mass in vivo, we generated transgenic mice expressing CrkII under the control of the alpha-1 type I collagen promoter. As shown in Fig. 7a, μCT analysis revealed a significant decrease in bone mass in CrkII transgenic mice compared with that in their wild-type littermates. The trabecular bone volume, thickness, and number were significantly decreased, whereas trabecular bone separation was significantly increased in CrkII transgenic mice compared with those in wild-type mice (Fig. 7b). In addition, the number of osteoblasts per bone perimeter was decreased in CrkII transgenic mice (Fig. 7c, d). Therefore, these results indicated that CrkII negatively regulates osteoblast differentiation and function in vivo.

## Discussion

Bone homeostasis is maintained by a constant balance between the activity of bone-resorbing osteoclasts and bone-forming osteoblasts. An imbalance between old bone resorption and new bone formation results in abnormal bone remodeling and the development of bone diseases, such as osteoporosis, Paget’s disease, and rheumatoid arthritis^39^. Many drugs and agents that either inhibit bone resorption or promote bone formation have been proposed for the treatment of bone diseases. Because bone remodeling is a coupled process between bone resorption and bone formation, there are certain limitations regarding drugs and agents targeting only one type of bone cell (osteoclast or osteoblast). For example, bisphosphonates, which are typical antiresorptive agents, effectively inhibit bone resorption but simultaneously inhibit bone formation, while parathyroid hormone, an anabolic agent, increases bone formation but also increases bone resorption^40–43^. Although our previous study showed that CrkII plays an important role in osteoclast differentiation and function, the role of CrkII in osteoblast differentiation and function remains unknown. To determine whether CrkII can simultaneously regulate bone resorption and bone formation, in the present study, we investigated the role of CrkII in osteoblast differentiation and function.

Our results evidently showed the negative role of CrkII in osteoblast differentiation and function. Overexpression of CrkII inhibited ALP activity, bone mineralization, and the expression of osteogenic marker genes, whereas downregulation of CrkII expression increased these factors. Interestingly, CrkII promoted JNK activation in osteoblasts, and inhibition of JNK activity by a JNK inhibitor completely reversed the inhibitory effects of CrkII on ALP activity, bone mineralization, and expression of osteogenic marker genes. Taken together, these results suggest that CrkII plays a negative role in osteoblast differentiation and function via the activation of JNK.

Although JNK is activated during osteoblast differentiation, its exact role in osteoblasts is somewhat controversial. JNK2 activation during osteoblast differentiation has been reported to be essential for late-stage differentiation in preosteoblast cells^44^. In contrast, activation of JNK has been shown to contribute to the inhibitory effects of tumor necrosis factor-α or TGF-β on osteoblast differentiation^45–47^. Notably, Huang et al.^6^ recently reported that JNK1 negatively regulates osteoblast differentiation via the phosphorylation of Runx2 on Ser104. That CrkII overexpression strongly phosphorylated JNK in osteoblasts and the blockade of JNK activation enhanced osteoblast differentiation and function inhibited by CrkII, even though the JNK pathway is strongly activated during osteoblast differentiation, suggests that activated JNK acts as an inhibitory factor for osteoblast differentiation.

Several studies have reported that Crk connects multiple cellular stimuli to the JNK signaling pathway. The JNK pathway was previously shown to be activated in v-Crk-transformed cells and cells transiently overexpressing CrkII^48,49^. Moreover, a direct interaction between JNK1 and CrkII was shown to be critical for JNK activation^50^. We found that CrkII connects osteogenic stimuli to the JNK pathway probably through binding with JNK1 (data not shown). Although CrkII phosphorylated both JNK1 and JNK2 in osteoblasts, its effect on the activation of JNK1 was stronger than that on the activation of JNK2. Huang et al.^6^ showed that JNK1 activation was strongly increased after BMP2 stimulation in multipotent C2C12 cells and preosteoblast MC3T3 cells, whereas JNK2 was activated to a lesser extent by BMP2 than JNK1. Furthermore, the CrkII-binding domain is conserved in JNK1 but not JNK2, and CrkII interacts only weakly with JNK2^50^. Collectively, these data support the possibility that the negative role of CrkII in osteoblast differentiation and function is linked to the activation of JNK1 rather than JNK2 via the direct interaction of CrkII with JNK1.

We showed here that the three SH domains in CrkII are required for CrkII-mediated osteoblast differentiation and function. Although the interaction between CrkII and JNK1 occurs through the N-terminal SH3 domain of CrkII, interestingly, JNK1 activation is significantly reduced by CrkII mutated in its SH2 domain compared to its activation by wild-type CrkII^50^. Since CrkII lacks a kinase domain, the optimal activation of JNK1 by CrkII likely requires the recruitment of several kinases, which may occur through the SH2 domain or C-terminal SH3 domain of CrkII, and the direct binding of CrkII with JNK1. For example, p130Cas, an adaptor protein containing several phosphorylatable tyrosine residues, is involved in JNK activation through the interaction of p130Cas with the SH2 domain of CrkII^50^. Since the present data strongly suggest that the function of CrkII in osteoblasts is linked to JNK activation, other domains in CrkII besides the N-terminal SH3 domain might contribute to JNK activation to regulate osteoblast differentiation and function.

We have previously reported that the role of CrkII in osteoclasts is linked to Rac1 activation^23^. Inhibition of Rac1 was shown to promote osteoblast differentiation upon BMP2 stimulation^38^. In the present study, we investigated whether the function of CrkII in osteoblasts is correlated with Rac1 activation, as it is in osteoclasts. Treatment with a Rac1 inhibitor partially reversed the inhibitory effects of CrkII on osteoblast differentiation, including its effects on ALP activity and the expression of Runx2 and ALP. However, the effects of the simultaneous inhibition of JNK and Rac1 were not synergistic, most likely due to the cytotoxic effects of pharmacological inhibitors (data not shown). Moreover, the inhibition of Rac1 did not affect JNK activation in osteoblasts overexpressing CrkII (data not shown). These results collectively suggest that Rac1 participates in CrkII-regulated osteoblast differentiation independent of the JNK signaling pathway. However, since several lines of evidence suggest that Rac1 is essential for CrkII-induced JNK activation^49^, we cannot exclude the possibility that Rac1 function in osteoblasts is closely related to JNK activation. Constitutively active Rac1 readily causes JNK activation, whereas a dominant negative form of Rac (Rac1N17) completely blocked Crk-induced JNK activation^49^. Collectively, these results suggest that Rac1 activation is a necessary step connecting Crk to JNK activation. Therefore, it is possible that the Rac1 inhibitor used in our experiments did not completely block Rac1 activity or that JNK was activated through the Ras pathway, an alternative pathway involving Crk-induced JNK activation, rather than the Rac1 pathway. It is necessary to identify whether Rac1 is involved in connecting CrkII to JNK activation in osteoblasts. Our future studies will investigate this issue.

In summary, our results showed that CrkII negatively regulates osteoblast differentiation and function through JNK activation, which reduces the expression of Runx2. Since CrkII also acts as a positive regulator of osteoclast differentiation and function, CrkII can regulate bone resorption as well as bone formation. Therefore, CrkII may serve as a new therapeutic target for the treatment of bone diseases by increasing bone formation and simultaneously inhibiting bone resorption.

## Supplementary information

Supplementary Figure
