# Supplementary material for: Adaptor protein CrkII negatively regulates osteoblast differentiation and function through JNK phosphorylation
Source: Exp Mol Med. 2019 Sep 25;51(9):111. doi: 10.1038/s12276-019-0314-3 (PMC6802640; doi:10.1038/s12276-019-0314-3)
Supplement: Supplementary file 1 — Supplementary Figure [file 12276_2019_314_MOESM1_ESM.doc]

**
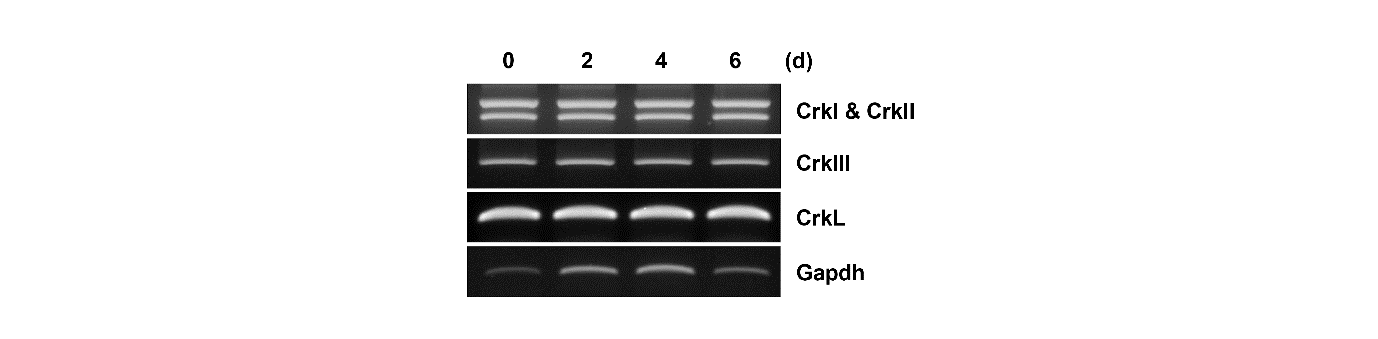
**

**Supplementary Fig. S1 Expression of CrkII during osteoblast differentiation.** Osteoblasts were cultured in OGM containing BMP2, ascorbic acid, and β-glycerophosphate for the indicated times. Total RNA was collected at each time point and RT-PCR was performed to assess the expression of the indicated genes.


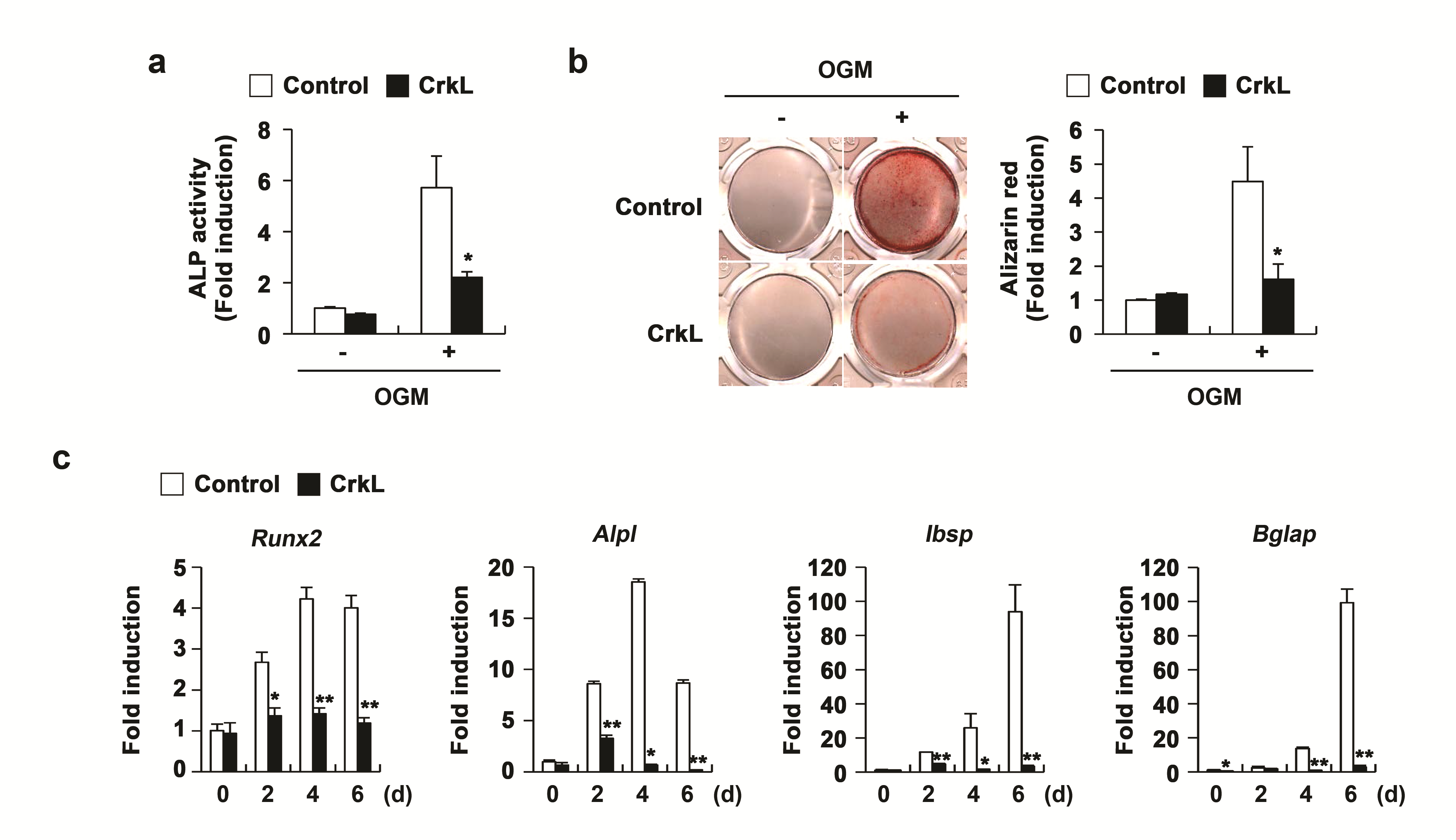


**Supplementary Fig. S2 CrkL overexpression inhibits osteoblast differentiation and function.** (a–c) Primary osteoblast precursor cells were transduced with pMX-IRES-EGFP (control) or CrkL retrovirus and cultured in OGM. (a) Cells were cultured for 3 days and subjected to ALP activity assay. (b) Cells were cultured for 9 days and fixed and stained with alizarin red (left panel). Staining intensities were quantified at 562 nm via densitometry (right panel). (c) Total RNA was collected at each indicated time point and real-time PCR was performed to evaluate the expression of the target genes. Data are expressed as the mean ± SD of triplicate samples. **p* < 0.01, ***p* < 0.001 versus control.


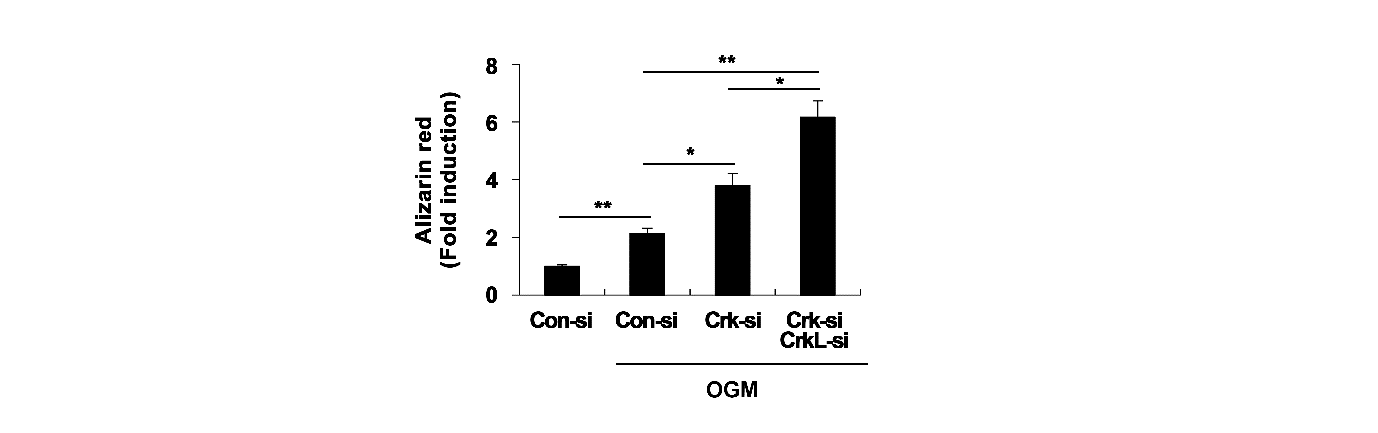
**Supplementary Fig.** **S3 CrkII and CrkL have overlapping function in osteoblasts.** Osteoblasts were transfected with control, Crk, or CrkL siRNA, as indicated. Cells were then cultured in OGM for 9 days and fixed and stained with alizarin red. Staining intensities were quantified at 562 nm via densitometry. Data are expressed as the mean ± SD of triplicate samples. **p* < 0.01, ***p* < 0.001 versus control.


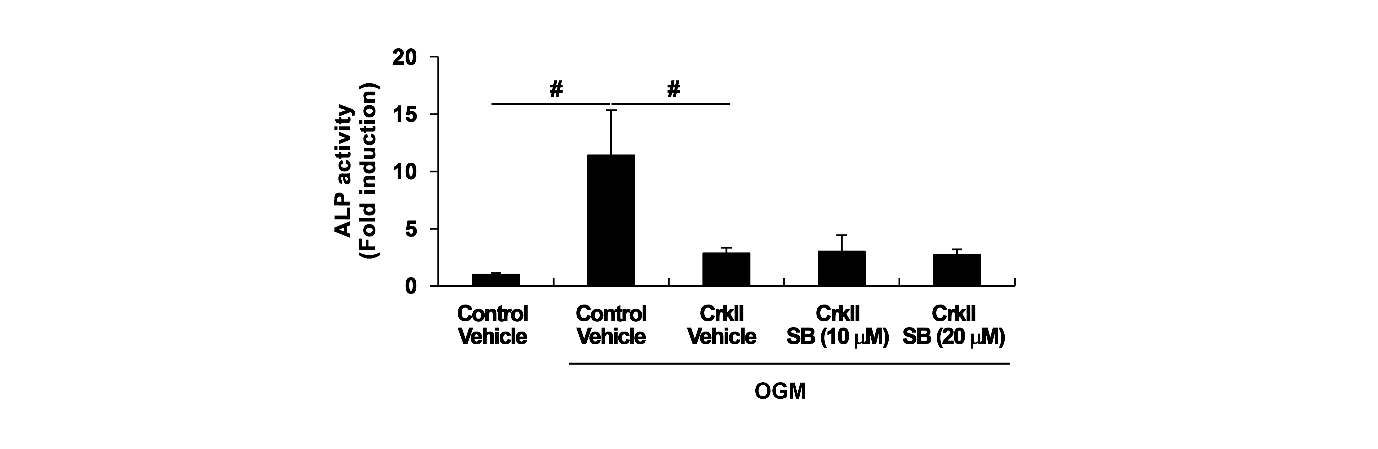


**Supplementary Fig. S4 CrkII regulates osteoblast differentiation independent of the p38 signaling pathway.** Osteoblasts were transduced with pMX-IRES-EGFP (control) or CrkII retrovirus. Transduced osteoblasts were treated with vehicle or SB203580, cultured in OGM for 3 days, and then subjected to ALP activity assay. Data are expressed as the mean ± SD of triplicate samples. #*p* < 0.05 versus control.
